# Silencing circular RNA-friend leukemia virus integration 1 restrained malignancy of CC cells and oxaliplatin resistance by disturbing dyskeratosis congenita 1

**DOI:** 10.1515/biol-2022-0036

**Published:** 2022-05-18

**Authors:** Weipeng Liu, Hong Jiang, Yuanqiang Li

**Affiliations:** Department of Gastrointestinal Surgery, The First College of Clinical Medical Science, China Three Gorges University, Phase 3, Jiangshan Duojiao, Wujiagang District, Yichang City, Hubei, 443000, China

**Keywords:** circ-FLI1, miR-197-3p, DKC1, CC

## Abstract

Circular-RNA friend leukemia virus integration 1 (circ-FLI1; hsa_circ_0000370) is a noninvasive biomarker for the diagnosis of colon carcinoma (CC). Herein, we intended to investigate its functions and competing endogenous RNA (ceRNA) mechanisms in CC cells. In terms of expression status, circ-FLI1 was abnormally upregulated in CC patients’ tumors and cells, paralleled with DKC1 upregulation and miR-197-3p downregulation. Most strikingly, there was a direct target relationship between miR-197-3p and circ-FLI1 or DKC1 based on the starbase database, dual-luciferase reporter assay, and RNA immunoprecipitation. Functionally, the colony formation assay, MTS method, fluorescence-activated cell sorting method, cell cycle and apoptosis assays, and transwell assays were performed, and the results revealed that interfering circ-FLI1 and re-expressing miR-197-3p could restrict colony formation, cell viability, cell cycle progression, and migration/invasion of CC cells with apoptosis rate elevation; besides, they promoted oxaliplatin (L-OHP)-induced cell viability inhibition. Furthermore, there were counteractive effects between circ-FLI1 silencing and miR-197-3p depletion, miR-197-3p overexpression and DKC1 restoration on regulating CC cell functions and L-OHP resistance. With a xenograft tumor model, the anti-growth role of circ-FLI1 silencing was also found *in vivo* with or without L-OHP treatment. Collectively, we demonstrated that circ-FLI1 might confer L-OHP resistance and malignant progression of CC presumably through the circ-FLI1/miR-197-3p/DKC1 ceRNA axis.

## 1 Introduction

In 2021, colon carcinoma (CC) was the most common fatal type of malignancy in men and women [1], and its incidence has been increasing in young and middle-aged people [2]. Metastasis is a fatal feature and major cause that leads to poor outcomes and cancer mortality [3]. Noncoding RNAs play a critical role in CC progression, CC treatment, and liver metastasis [4,5].

Circular RNAs (circRNAs) are a very interesting class of noncoding RNA molecules derived from precursor messenger RNA (mRNA) via back-splicing events. CircRNA expression seems to be abundant and stable with tissue- and developmental stage-specificity [6]. In CC, circRNAs are aberrantly expressed in different types of human samples and could mediate and predict tumorigenesis, metastasis, and chemoradiation resistance [7]. Hsa_circ_0000370 is a loop structure with 637 bp derived from the friend leukemia virus integration 1 (FLI1) gene located on chr11:128628009-128651918, hereafter termed as circ-FLI1; circ-FLI1 is dysregulated in human CC plasma and could be a noninvasive biomarker for the diagnosis of CC [8]. However, this circRNA has not been comprehensively investigated in CC-related proliferation and metastasis.

Dyskeratosis congenit 1 (DKC1) is a tumor suppressor, and its gene mutation could cause cancer-prone syndrome [9]; DKC1 regulates CC angiogenesis and metastasis [10]. DKC1-encoded dyskerin could directly bind to several microRNAs (miRNAs) embedded in H/ACA ribonucleoproteins [11], and DKC1 depletion reduces the accumulation of a subset of miRNAs [12]. However, DKC1 has not been functionally validated in CC. miRNA (miR)-197-3p is extensively studied in different cancer carcinogenesis progression via serving as an oncogene or anti-oncogene [13]. In CC, miR-197-3p has been demonstrated to be downregulated in CC specimens and suppresses CC development and chemoresistance [14,15].

Oxaliplatin (L-OHP)-based chemotherapy combination is the common first-line chemotherapeutic regimen for metastatic CC [16], and its resistance is a major reason for treatment failure [17]. Therefore, our aim is to investigate circ-FLI1 functions in malignant behaviors and L-OHP resistance in CC cells and to probe into its underlying molecular mechanism by regulating miR-197-3p and DKC1.

## 2 Materials and methods

### 2.1 Patients and cells

A total of 56 CC primary patients were enrolled from The First College of Clinical Medical Science, China Three Gorges University, between 06/2015 and 12/2020. Clinicopathological characteristics of these 56 patients are shown in Table A1. The inclusion criteria are as follows: patients were allowed to collect the tumor tissues and adjacent (>3 cm) normal tissues and patients received the FOLFOX regimen after the surgery. Tumor and normal tissues were confirmed using immunohistochemistry. The exclusion criteria are as follows: patients with other tumors, patients with autoimmune diseases, and patients with CC family history. Overall survival was obtained from the postoperative follow-up for 5 years, and overall survival time was defined as the time from surgery to death or the last follow-up.

Four CC cell lines HCT116 (#91091005), SW480 (#87092801), SW620 (#87051203), and LoVo (#87060101) were collected from ECACC (Salisbury, UK), and one normal colonic epithelial cell line NCM460 (#75) was collected from LONZA (Basel, Switzerland). All cell lines were normally cultured according to the suggestion.


**Informed consent:** Informed consent was obtained from all individuals included in this study.
**Ethical approval:** The research related to human use has been complied with all the relevant national regulations, and institutional policy is in accordance with the tenets of the Helsinki Declaration and has been approved by the authors’ institutional review board or equivalent committee.

### 2.2 Cell transfection

circ-FLI1, miR-197-3p, and DKC1 expressions were modified by transfecting exogenous nucleotides via Lipofectamine 2000 Reagent (Invitrogen). circ-FLI1 was silenced by transfecting its siRNAs (si-circ-FLI1#1 ACAGAGCCUCCUUCUUUGACAdTdT and si-circ-FLI1#2 AGAGCCUCCUUCUUUGACACUdTdT) or transfecting pSilencer2.1-U6 hygro (EK-Bioscience, Shanghai, China) vectors carrying its shRNA (sh-circ-FLI1 AGCTACAGAGCCTCCTTCTTTGACATCAAGAGTGTCAAAGAAGGAGGCTCTGTTTTTTG and GATCCAAAAAACAGAGCCTCCTTCTTTGACACTCTTGATGTCAAAGAAGGAGGCTCTGT); circ-FLI1 was overexpressed by transfecting a recombinant vector (pCD-ciR-circ-FLI1, circ-FLI1). miR-197-3p gene manipulation was done using a commercial mimic or inhibitor. DKC1 was overexpressed using a recombinant vector (pcDNA-DKC1, DKC1). Transfected cells at 48 h were harvested for further analysis.

### 2.3 Real-time quantitative polymerase chain reaction (RT-qPCR)

Total RNAs in cells and tissues were extracted using the RNAsimple total RNA kit (Tiangen, Beijing, China), and 0.5 µg of RNA was then reverse transcribed to the first strand of cDNA using the high capacity cDNA reverse transcription kit (Applied Biosystems, Foster City, CA, USA). Eventually, real-time quantification of cDNA was launched using SYBR Green qPCR Master Mix (Invitrogen). The All-in-One miRNA first-strand cDNA synthesis kit (GeneCopoeia, Rockville, MD, USA) and All-in-One miRNA qRT-PCR detection kit (GeneCopoeia) were used to miRNA expression. The special RT-qPCR primers for circ-FLI1, FLI1, miR-197-3p, DKC1, U6, and glyceraldehyde-phosphate dehydrogenase (GAPDH) are shown in Table A2. Relative RNA expression of circ-FLI1, FLI1, miR-197-3p, and DKC1 was calculated by the cycle threshold method and corrected by internal control GAPDH or U6.

### 2.4 RNase R treatment and subcellular localization analysis

For RNase R treatment, total RNAs (2 µg) from HCT116 and SW480 cells were exposed with 5U RNase R (Geneseed, Guangzhou, China) or an equal volume of the 1× reaction buffer (Geneseed) for 30 min at 37°C; then, RNase R was inactivated at 70°C for 10 min, and RNase R/mock-treated RNAs were subjected to RT-qPCR analysis. For subcellular localization analysis, the Nuclear/Cytosol Fractionation kit (BIOMARS, Beijing, China) was used to separate the nuclear extract and cytoplasmic fraction from HCT116 and SW480 cells, followed by RT-qPCR analysis.

### 2.5 Colony formation assay

Transfected HCT116 and SW480 cells were inoculated in a 12-well plate at a density of 200 cells per well. These cells were cultured in the normal medium for 2 weeks with medium change every 3 days. Afterward, colonies were formed and shown with the crystal violet staining method. The number of colonies (more than 50 cells) was recorded.

### 2.6 Cell viability assay

Cell viability of transfected HCT116 and SW480 cells for continuous 3 days was monitored and measured with the MTS method with the aid of the CellTiter 96 Aqueous Non-Radioactive Cell Proliferation Assay kit (Promega, Madison, WI, USA). In brief, cells were plated in 96-well plates at a density of 3 × 10^3^ cells per well, and 10 µL of MTS reagent was added to each well at 0, 24, 48, and 72 h after cell adherence. The absorbance at 495 nm was determined on a microplate reader.

### 2.7 Cell cycle analysis and apoptosis assay

Annexin V fluorescein isothiocyanate (FITC) Apoptosis Detection kit (Vazyme, Nanjing, China) and fluorescence-activated cell sorting (FACS) method were combined to measure the apoptosis rate. In brief, transfected HCT116 and SW480 cells were subjected to FITC-labeled Annexin V (Annexin V-FITC) and propidium iodide (PI) counterstain, and stained cells were sorted in the Annexin V-FITC/PI quadrant. For cell cycle analysis, transfected cells were fixed with cold 75% ethanol and stained with PI staining buffer containing 500 µL of binding buffer (Beyotime), 25 µL of 20× PI (Beyotime, Shanghai, China), and 10 µL of 50× RNase A (Beyotime). Cells in different cell cycle phases were analyzed on a flow cytometer (BD Biosciences, Franklin Lakes, NJ, USA) equipped with Cell Quest software (BD Biosciences).

### 2.8 Transwell assays

Transfected HCT116 and SW480 cells were collected and resuspended in a serum-free medium. Then, 2 × 10^4^ cells were inoculated in the upper chamber, and the lower chamber in a 24-well plate was filled with the complete culture medium (containing 10% fetal bovine serum). This system was incubated under normal cell culture conditions for 24 h, and transferred cells onto the lower surface of chamber were shown with the crystal violet staining method. For the migration assay, the Transwell chamber (Corning, New York, NY, USA) was used; for the invasion assay, the Transwell chamber (Corning) was equipped with Matrigel (BD Biosciences) by incubating the chamber in Matrigel:medium (1:9) at 37°C for 4 h. The numbers of migrated cells and invaded cells per field were counted under a microscope at 100×.

### 2.9 Dual-luciferase reporter assay

Online circinteractome, starbase, Targetscan, MicroT-CDS, and TarBase databases were used to predict the miRNA-binding site, and Venn diagram was used to analyze the overlapping results based on two or multiple databases. According to the starbase database, the putative intact miR-197-3p-binding sites in the sequence of circ-FLI1 and DKC1 3′-UTR were mutated. Then, the wild type (WT) of circ-FLI1 and DKC1 3′-UTR were cloned into a pGL4 reporter vector (Promega, Madison, WI, USA). In addition, mutant types (MUTs) of them were generated by performing site-directed mutation based on WT reporter vectors and via the QuickChange Lightning multisite-directed mutagenesis kit (Stratagene, Cedar Creek, TX, USA). These recombinant vectors (100 ng) were co-transfected with 50 nM miR-197-3p mimic (miR-197-3p) or miR-NC mimic (miR-NC) in HCT116 and SW480 cells in a 96-well plate for 48 h. The luciferase activities were detected using the Dual-Luciferase Reporter Assay System (Promega) on a GloMax-20/20 luminescence detector (Promega).

### 2.10 RNA immunoprecipitation (RIP)

Cell lysates of HCT116 and SW480 cells were harvested for IgG RIP using the RIP assay kit (MBL, Woburn, MA, USA). For argonaute 2 (AGO2) RIP, anti-Ago2 (ab32381; Abcam, Cambridge, UK) and Protein A/G Agarose beads (Pierce, Rockford, IL, USA) were purchased; the cell lysate was incubated with the beads precoupled with anti-AGO2 at 4°C for 12 h. Eventually, beads were eluted and digested with proteinase K, and immunoprecipitated RNAs were analyzed on RT-qPCR.

### 2.11 Western blotting

Total Protein Extraction kit (Invent Biotechnologies, Plymouth, MN, USA) was used to isolate total protein from tissues or cells. In total, 25 µg of total proteins was suspended in a 5× sodium dodecyl sulfate–polyacrylamide gel electrophoresis (SDS–PAGE) sample loading buffer (Beyotime) and boiled at 90°C for 5 min. Next, SDS–PAGE and membrane transfer were sequentially performed prior to antibody incubation for DKC1 (sc-373956, 1:500, Santa Cruz Biotechnology, Shanghai, China), GAPDH (AF5009, 1:5,000; Beyotime), and mouse IgG(H + L) (A0216, 1:1,000; Beyotime). The signals were detected by the ECL-PLUS kit (GE Healthcare, Piscataway, NJ, USA) and MYECL Imager (Thermo Fisher Scientific). Relative DKC1 protein expression was calculated with normalization to GAPDH.

### 2.12 Tumorigenicity model assay

A total of 24 male nude mice (BALB/C; Vital River Laboratory, Beijing, China) were seeded for the xenograft experiment. HCT116 cells were stably transfected with sh-circ-FLI1 or sh-NC vectors before the xenograft experiment. For the xenograft tumor model assay, cell suspension of stably transfected HCT116 cells (1 × 10^6^ cells/mouse) was subcutaneously injected into the right anterior axilla, and 12 mice were set per group. After cell injection for 4 days, mice were intraperitoneally injected with L-OHP (6 mg/kg body weight) or an equal volume of phosphate-buffered saline (PBS) with four mice per group. L-OHP/PBS treatment was performed every 4 days for six times. Tumor volume was monitored every 4 days after cell inoculation and calculated by 0.5 × length × width^2^. Tumor-bearing mice were raised for 4 weeks and then euthanized. Xenograft tumors were excised, imaged, weighed, and stored.


**Ethical approval:** The research related to animal use has been complied with all the relevant national regulations and institutional policies for the care and use of animals, and was approved by the Ethics Committee of The First College of Clinical Medical Science, China, Three Gorges University. Animal care was in accordance with the Provision and General Recommendation of Chinese Experimental Animals Administration Legislation.

### 2.13 Statistical analysis

All experiments were performed three independent times, and data were expressed as mean ± standard deviation. Data were statistically analyzed using Welch’s *t*-test and one-way/two-way analysis of variance (ANOVA). Tukey’s/Sidak’s/Dunnett’s *post hoc* test was used following ANOVA. Data analysis was performed on GraphPad Prism7 (GraphPad, La Jolla, CA, USA). The correlation between circ-FLI1 and clinicopathological characteristics of these 56 patients is shown in Table A1. The Kaplan–Meier survival curve and log-rank (Mantel–Cox) test displayed the correlation between circ-FLI1 expression and overall survival of this cohort of 56 CC patients.

## 3 Results

### 3.1 circ-FLI1 was upregulated in CC

**Figure 1:**
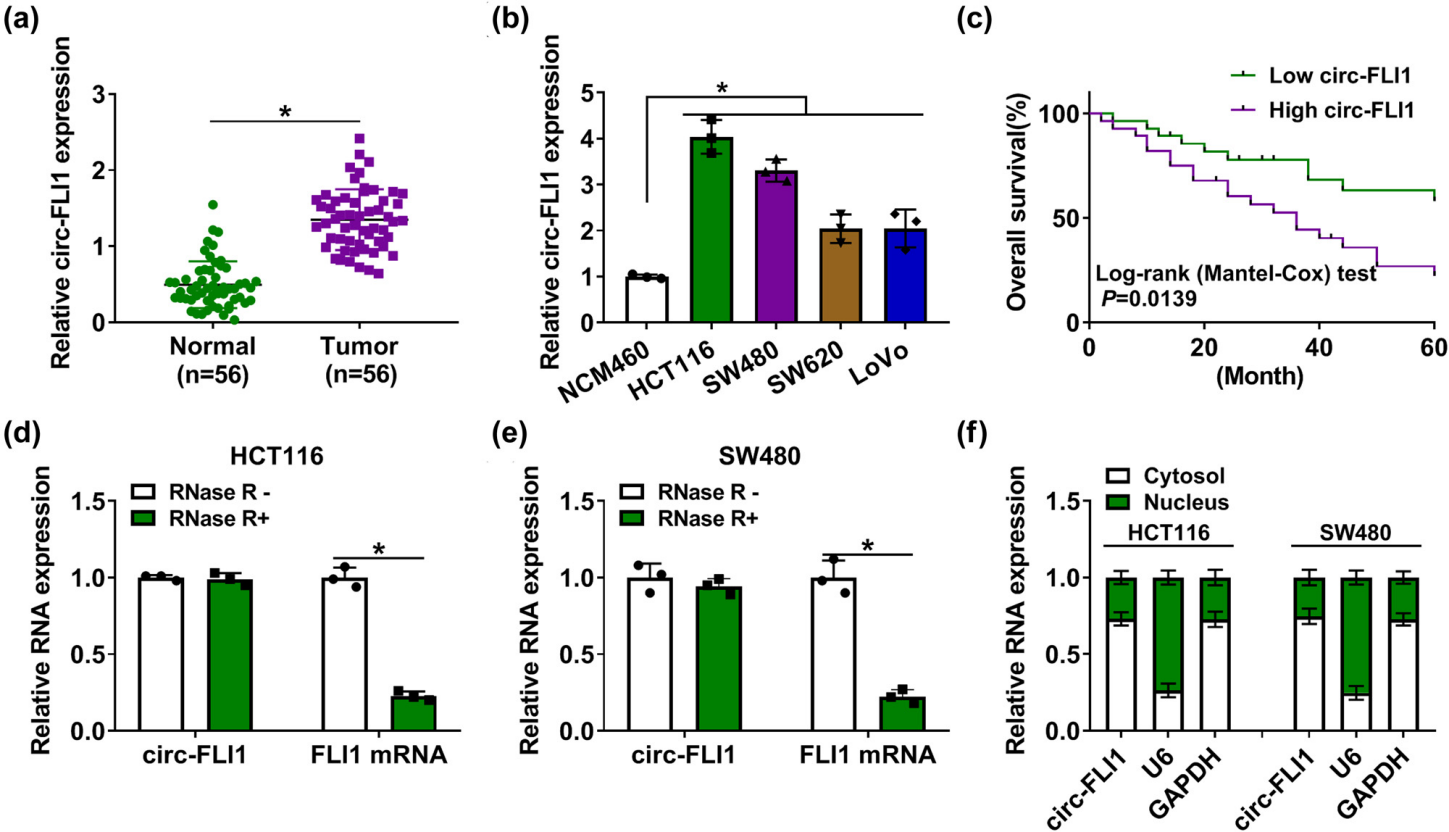
circ-FLI1 was upregulated in CC and might predict poor prognosis. (a and b) RT-qPCR detected relative cir-FLI1 expression in (a) tissue samples in normal and tumor groups from 56 CC patients and (b) cell samples in NCM460, HCT116, SW480, SW620, and LoVo groups. (c) Kaplan–Meier survival curve and log-rank (Mantel–Cox) test analyzed the association between circ-FLI1 level and overall survival of 56 CC patients divided into high circ-FLI1 group (*n* = 28) and low circ-FLI1 group (*n* = 28). (d–f) RT-qPCR detected relative RNA expression of circ-FLI1, FLI1 mRNA, GAPDH, or U6 in (d and e) total RNAs in RNase R + and RNase R-groups and (f) cytosol RNAs and nuclear RNAs in HCT116 and SW480 cells. *P < 0.05.

RT-qPCR data showed a high level of circ-FLI1 in CC tumor tissues than the normal tissues (Figure 1a); its expression was overall upregulated in CC cell lines versus normal cell lines (Figure 1b). Moreover, the Kaplan–Meier survival curve and log-rank (Mantel–Cox) test displayed a negative correlation between circ-FLI1 expression and overall survival in these 56 CC patients (Figure 1c); moreover, high circ-FLI1 showed a significant correlation with tumor size, TNM stage, and distant metastasis in these CC patients (Table A1). Compared with the linear counterpart, circ-FLI1 expression in HCT116 and SW480 cells was resistant to RNase R treatment (Figure 1d and e). In addition, circ-FLI1 allied with cytoplasmic control GAPDH was mostly detected in the cytosol (Figure 1f). These data depicted that circ-FLI1 was abnormally upregulated in human CC tissue and cell samples.

### 3.2 Interfering circ-FLI1 restrained malignant process of CC cells and L-OHP resistance

**Figure 2:**
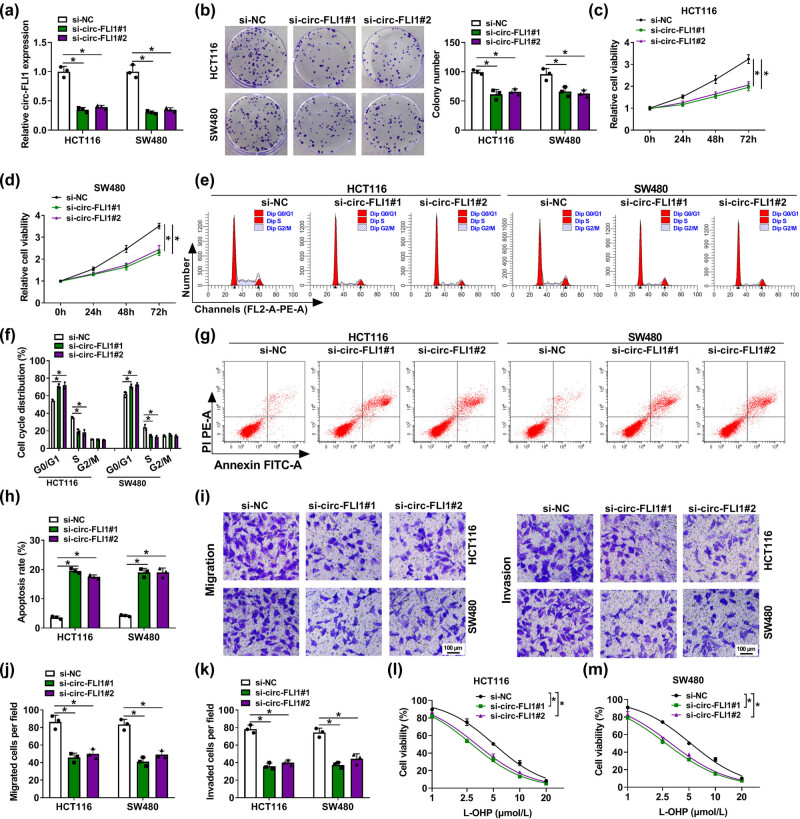
Interfering circ-FLI1 restrained malignant process of CC cells and L-OHP resistance. HCT116 and SW480 cells were transfected with si-circ-FLI1#1 or si-circ-FLI1#2 with normalization to si-NC transfection. (a) RT-qPCR detected relative circ-FLI1 expression. (b) Colony formation assay determined colony number. (c and d) Continuous monitoring of relative cell viability every 24 h in 3 days was performed by the MTS assay. (e and f) FACS and cell cycle assay kit evaluated cell cycle distribution in G0/G1, S, and G2/M phases. (g and h) FACS and apoptosis assay kit examined apoptosis rate. (i–k) Transwell assays measured cell migration and invasion, and numbers of migrated cells and invaded cells per field (100×) were counted. (l and m) MTS assay monitored relative cell viability with different concentrations of L-OHP treatment. *P < 0.05.

**Figure A1:**
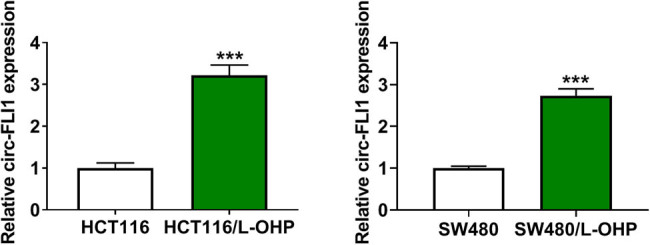
circ-FLI1 was upregulated in L-OHP-resistant CC cells. RT-qPCR detected relative circ-FLI1 expression in SW480/L-OHP and HCT116/L-OHP cells compared with the parental SW480 and HCT116 cells. ***P < 0.001.

Either si-circ-FLI1#1 or si-circ-FLI1#2 transfection could mediate the silencing of circ-FLI1 in HCT116 and SW480 cells (Figure 2a), which weakens colony formation ability (Figure 2b). Besides, the MTS cell viability of HCT116 and SW480 cells analyzed was inhibited in 3 days by interfering circ-FLI1 via siRNA (Figure 2c and d). In response to circ-FLI1 siRNAs transfection, FACS determined that cell cycle distribution was altered in HCT116 and SW480 cells, as evidenced by the increase of G0/G1 cells and the decrease of S cells (Figure 2e and f). Similarly, the apoptosis rate was highly induced in the presence of si-circ-FLI1#1 or si-circ-FLI1#2 (Figure 2g and h). Transwell migration/invasion capacities were both suppressed in circ-FLI1-silenced HCT116 and SW480 cells, as indicated by the lowered migrated cells and invaded cells per field (Figure 2i–k). Furthermore, circ-FLI1 expression was upregulated in L-OHP-resistant CC cells (Figure A1), and its silencing could promote L-OHP-induced cell viability inhibition in these parental cells (Figure 2l and m). These results concluded that inhibiting circ-FLI1 could restrain colony formation, cell proliferation, and migration/invasion of CC cells but promote cell cycle arrest, apoptosis, and L-OHP chemosensitivity.

### 3.3 miR-197-3p was a target for circ-FLI1

**Figure 3:**
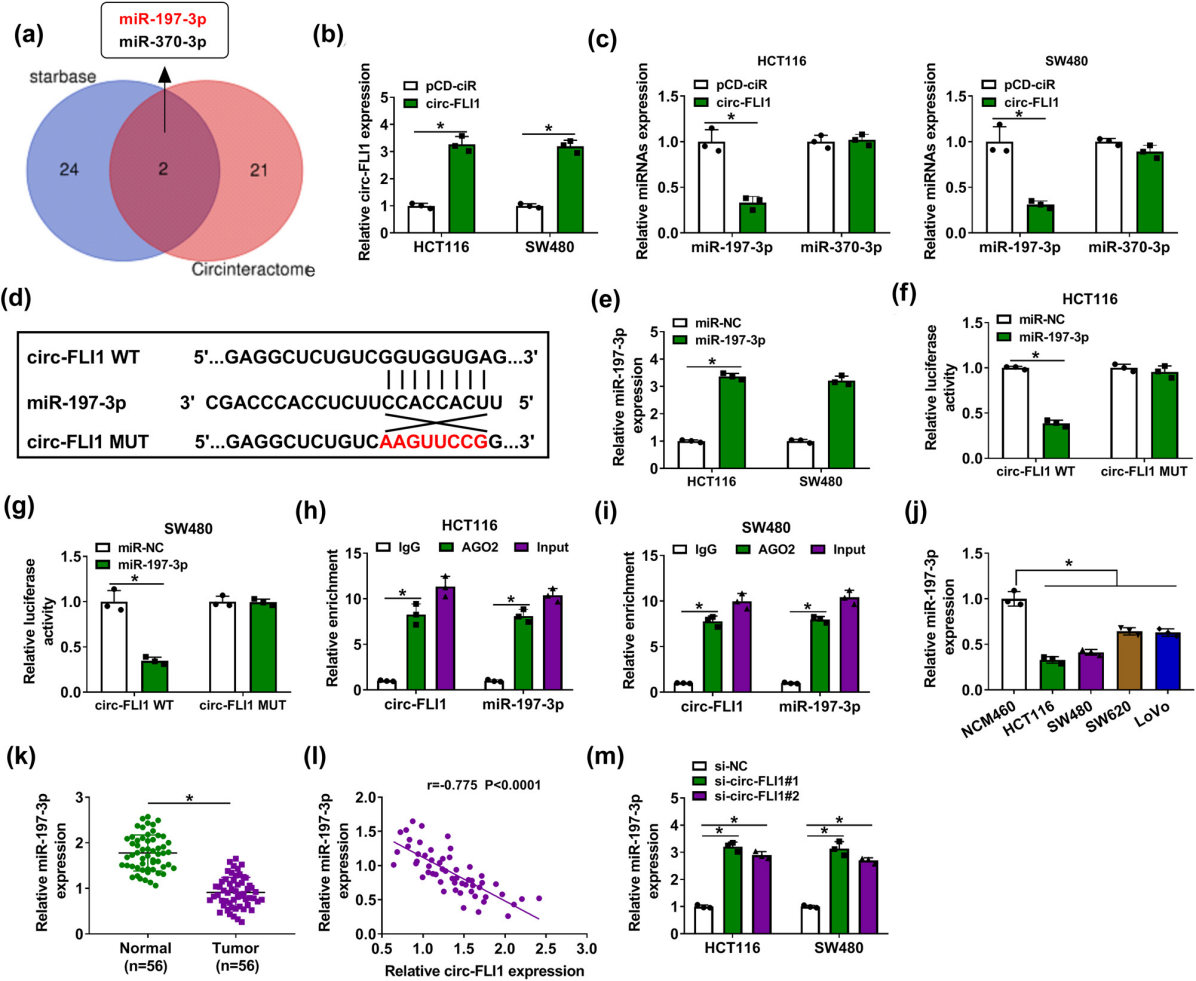
miR-197-3p was a target for circ-FLI1. (a) Venn diagram showed the overlapping results of starbase and circinteractome databases for miRNA binding prediction in the sequence of circ-FLI1. (b and c) RT-qPCR detected relative circ-FLI1, miR-197-3p and miR-370-3p expression in HCT116 and SW480 cells transfected with circ-FLI1 or pCD-ciR vector. (d) The alignment sequence of circ-FLI1 WT, miR-197-3p and circ-FLI1 MUT was presented. (e) RT-qPCR detected relative miR-197-3p expression in miR-197-3p-transfected HCT116 and SW480 cells with normalization to miR-NC transfection. (f and g) Dual-luciferase reporter assay measured relative luciferase activity of circ-FLI1 WT and circ-FLI1 MUT in miR-197-3p or miR-NC-transfected HCT116 and SW480 cells. (h and i) RIP assay identified relative enrichment of circ-FLI1 and miR-197-3p by AGO2 in HCT116 and SW480 cells with normalization to that by IgG. (j and k) RT-qPCR detected relative miR-197-3p expression in (j) tissue samples in Normal and Tumor groups from 56 CC patients and (k) cell samples in NCM460, HCT116, SW480, SW620 and LoVo groups. (l) Pearson’s correlation test validated the correlation between circ-FLI1 and miR-197-3p expression in 56 CC tumor tissues. (m) RT-qPCR detected relative miR-197-3p expression in HCT116 and SW480 cells transfected with si-circ-FLI1#1, si-circ-FLI1#2, or si-NC transfection. *P < 0.05.

Based on starbase and circinteractome predictions, miR-197-3p and miR-370-3p were the common computational targets of circ-FLI1 (Figure 3a), and only miR-197-3p expression was responsive to overexpression of circ-FLI1 via vector transfection (Figure 3b and c). For further validation, circ-FLI1 MUT was designed by mutating the predicted miR-197-3p-binding site (Figure 3d). miR-197-3p transfection led to the overexpression of miR-197-3p in HCT116 and SW480 cells (Figure 3e), and allied with this was the deficit of luciferase activity of reporter vector expressing circ-FLI1 WT and the stable luciferase activity of the MUT vector (Figure 3f and g). The AGO2 RIP assay tested a relative enrichment of circ-FLI1 and miR-197-3p in HCT116 and SW480 cells (Figure 3h and i). These assays identified a direct target relationship between circ-FLI1 and miR-197-3p. Moreover, the relative expression of miR-197-3p was abnormally decreased in human CC cell lines and tumor tissues than corresponding normal controls (Figure 3j and k). Accidently, miR-197-3p expression in CC patients’ tumors was significantly and negatively correlated with circ-FLI1 (Figure 3l). miR-197-3p expression was also upregulated by circ-FLI1 knockdown via siRNA transfection (Figure 3m). These data demonstrated that circ-FLI1 controlled the miR-197-3p level in CC via target binding.

### 3.4 Inhibiting miR-197-3p abated effects of circ-FLI1 interference on cell behavior of CC cells and chemoresistance to L-OHP

**Figure 4:**
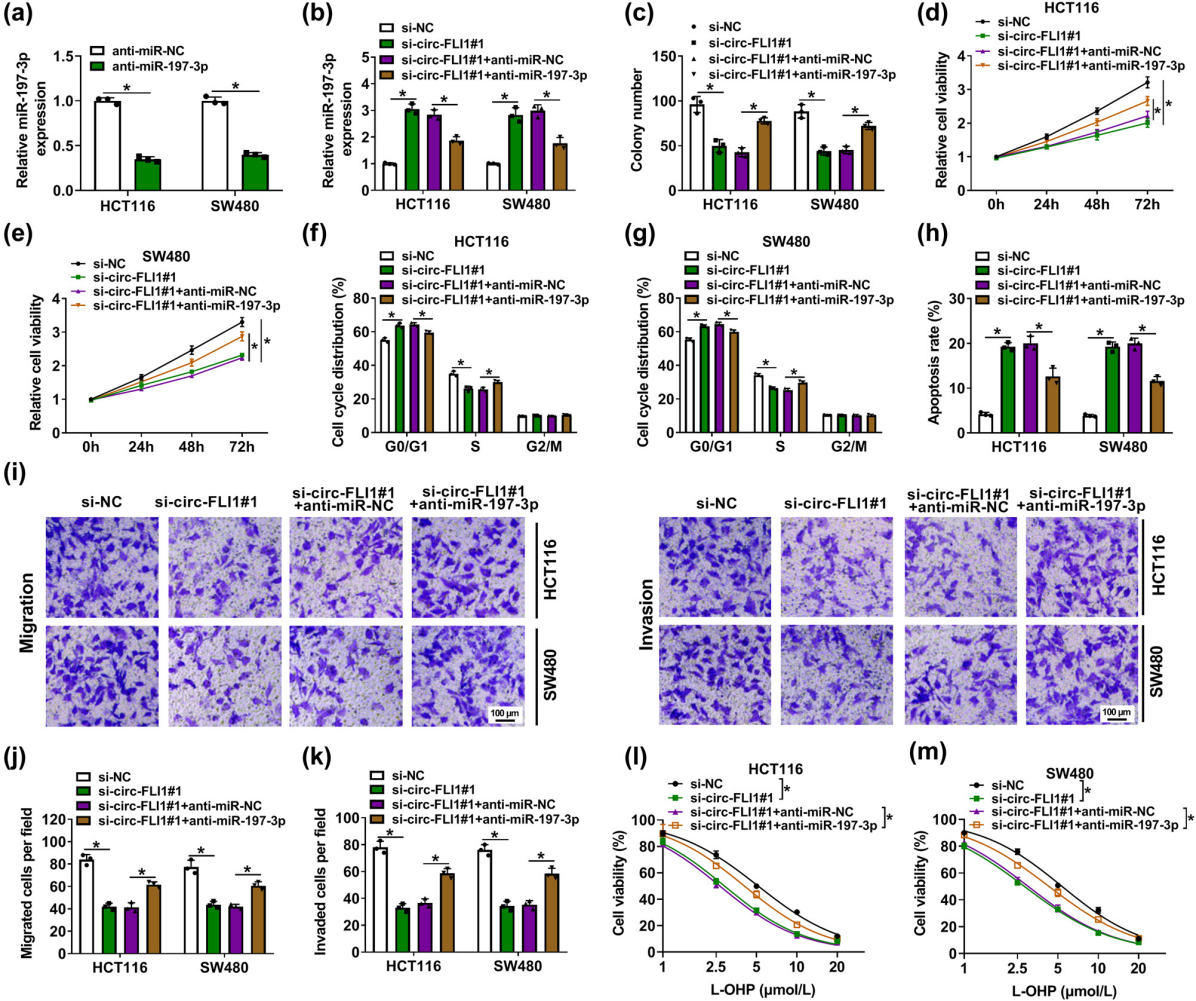
Inhibiting miR-197-3p abated effects of circ-FLI1 interference in CC cells. (a) RT-qPCR detected relative miR-197-3p expression in anti-miR-197-3p-transfected HCT116 and SW480 cells with normalization to anti-miR-NC transfection. (b–m) HCT116 and SW480 cells were transfected with si-circ-FLI1#1 alone or along with anti-miR-197-3p, normalized to si-NC transfection or si-circ-FLI1#1 and anti-miR-NC co-transfection. (b) RT-qPCR detected relative miR-197-3p expression. (c) Colony formation assay determined colony number. (d and e) MTS assay continuously monitored relative cell viability every 24 h in 3 days. (f and g) FACS and cell cycle assay kit evaluated cell cycle distribution in G0/G1, S, and G2/M phases. (h) FACS and apoptosis assay kit examined apoptosis rate. (i–k) Transwell assays measured cell migration and invasion, and numbers of migrated cells and invaded cells per field (100×) were counted. (l and m) MTS assay monitored relative cell viability with different concentrations of L-OHP treatment. *P < 0.05.

Transfecting miR-197-3p inhibitor (anti-miR-197-3p) resulted in the inhibition of miR-197-3p expression in HCT116 and SW480 cells as well as in circ-FLI1-silenced HCT116 and SW480 cells (Figure 4a and b). Allied with that, colony formation number and cell proliferation ability in circ-FLI1-downregulated cells were improved with the transfection of anti-miR-197-3p (Figure 4c–e). Cell cycle distribution in the S phase was lowered, and the apoptosis rate was facilitated by circ-FLI1 blockage, and these effects were partly abated by further blocking miR-197-3p (Figure 4f–h). Interfering circ-FLI1 restricted transwell migration/invasion in HCT116 and SW480 cells, which is restored with co-transfecting anti-miR-197-3p (Figure 4i–k). Finally, the suppressive effect of circ-FLI1 knockdown on cell viabilities of L-OHP-treated HCT116 and SW480 cells was distinctively attenuated by inhibiting miR-197-3p (Figure 4l and m). These results indicated that miR-197-3p inhibition could abate circ-FLI1 interference role in CC malignant progression and L-OHP resistance.

### 3.5 DKC1 was targeted by miR-197-3p

**Figure 5:**
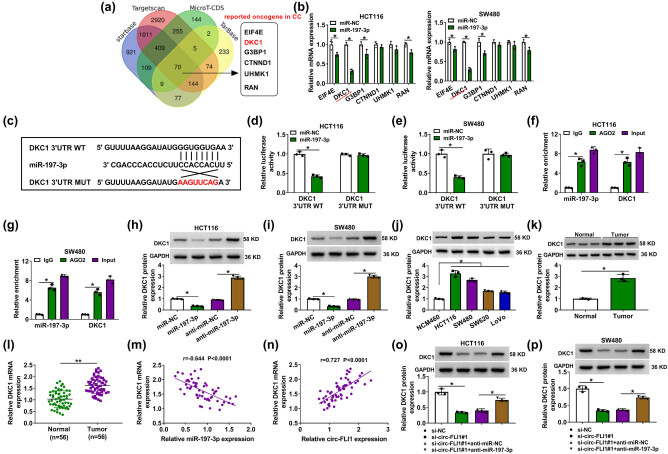
DKC1 was targeted by miR-197-3p. (a) Venn diagram showed the overlapping results of starbase, Targetscan, MicroT-CDS, and TarBase databases for miRNA binding prediction of miR-197-3p in mRNAs, and among them the six reported oncogenes in CC were showed. (b and c) RT-qPCR detected relative mRNA expression of EIF4E, DKC1, G3BP1, CTNND1, UHMK1, and RAN in HCT116 and SW480 cells transfected with miR-197-3p or miR-NC. (c) The alignment sequence of DKC1 3′-UTR WT, miR-197-3p, and DKC1 3′-UTR MUT was presented. (d and e) Dual-luciferase reporter assay measured relative luciferase activity of DKC1 3′-UTR WT and DKC1 3′-UTR MUT in miR-197-3p or miR-NC-transfected HCT116 and SW480 cells. (f and g) RIP assay identified relative enrichment of DKC1 and miR-197-3p by AGO2 or IgG in HCT116 and SW480 cells. (h–j) Western blotting detected relative DKC1 protein expression in (h and i) miR-NC, miR-197-3p, anti-miR-NC, or anti-miR-197-3p-transfected HCT116 and SW480 cells, and (j) NCM460, HCT116, SW480, SW620, and LoVo cells. (k and l) Western blotting and RT-qPCR severally detected relative DKC1 protein and mRNA expressions in tissue samples in normal and tumor groups from CC patients. (m and n) Pearson’s correlation test validated correlations between DKC1 mRNA and miR-197-3p or circ-FLI1 expression in 56 CC tumor tissues. (o and p) Western blotting detected relative DKC1 protein expression in si-NC or si-circ-FLI1#1-transfected and si-circ-FLI1#1 and anti-miR-197-3p or anti-miR-NC-co-transfected HCT116 and SW480 cells. *P < 0.05.

The Venn diagram showed a 70-mRNA signature of miR-197-3p target genes according to the starbase, Targetscan, MicroT-CDS, and TarBase databases; among them, there were six mRNAs (EIF4E [18], DKC1 [19], G3BP1 [20], CTNND1 [21], UHMK1 [22], and RAN [23]) that had been reported as oncogenes in CC (Figure 5a). Moreover, relative mRNA expressions of EIF4E, DKC1, G3BP1, and RAN were significantly downregulated by miR-197-3p overexpression (Figure 5b). Based on the starbase prediction result, there were three miR-197-3p-binding sites in DKC1, and the site at chrX: 154005323–154005345[+] was selected to construct DKC1 MUT via site-directed mutation at 3′-UTR (Figure 5c). miR-197-3p overexpression via mimic transfection could only reduce the luciferase activity of the DKC1 3′-UTR WT reporter vector instead of the MUT vector (Figure 5d and e). Moreover, miR-197-3p together with DKC1 could be enriched by AGO2 RIP in HCT116 and SW480 cells (Figure 5f and g). Western blotting data showed that DKC1 protein expression was inversely modulated by miR-197-3p (Figure 5h and i) and was highly expressed in human CC cell lines and tumor tissues (Figure 5j and k). Besides, relative DKC1 mRNA expression was increased in CC patients’ tumors (Figure 5l), and tumor tissue DKC1 mRNA expression was with negative correlation with miR-197-3p and positive correlation with circ-FLI1 (Figure 5m and n). Except for that, DKC1 protein level could be downregulated by circ-FLI1 silencing via siRNA transfection, and this downregulation was counteracted by blocking miR-197-3p via anti-miR-197-3p transfection (Figure 5o and p). These results demonstrated that DKC1 could be targeted by miR-197-3p, and its expression was regulated by circ-FLI1 via miR-197-3p.

### 3.6 Re-expressing miR-197-3p restricted malignant behavior and L-OHP resistance in CC cells via disturbing DCK1

**Figure 6:**
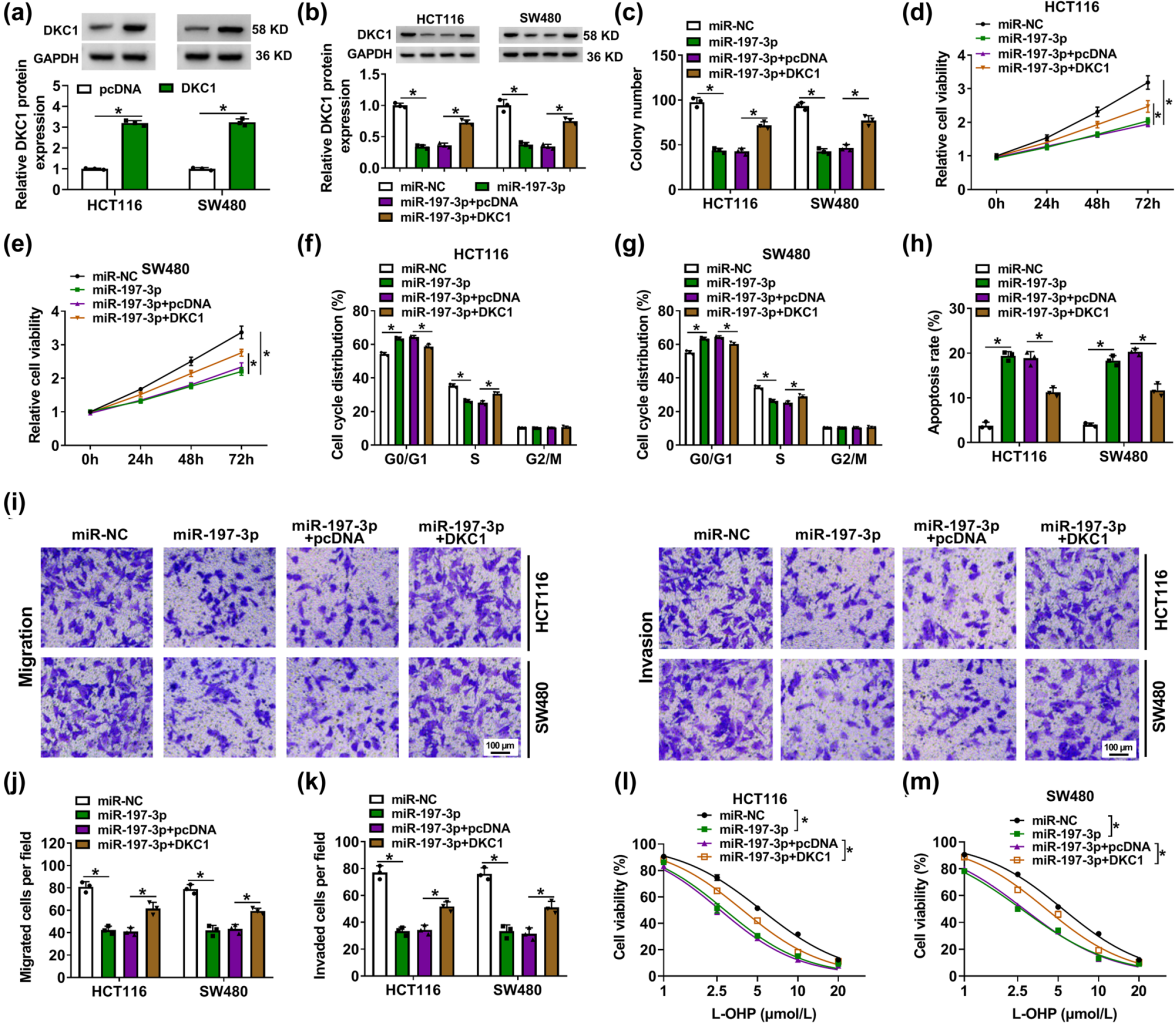
Re-expressing miR-197-3p restricted malignant behavior and L-OHP resistance in CC cells relying on DCK1 downregulation. (a and b) Western blotting detected relative DKC1 protein expression in HCT116 and SW480 cells transfected with DKC1, pcDNA vector, miR-NC, or miR-197-3p, and co-transfected with miR-197-3p and DKC1 or pcDNA vector. (c) Colony formation assay determined colony number. (d and e) MTS assay continuously monitored relative cell viability every 24 h in 3 days. (f and g) FACS and cell cycle assay kit evaluated cell cycle distribution in G0/G1, S, and G2/M phases. (h) FACS and apoptosis assay kit examined the apoptosis rate. (i–k) Transwell assays measured cell migration and invasion, and numbers of migrated cells and invaded cells per field (100×) were counted. (l and m) MTS assay monitored relative cell viability with different concentrations of L-OHP treatment. *P < 0.05.

DKC1 overexpression vector could reinforce DKC1 protein expression via transfection in HCT116 and SW480 cells and in miR-197-3p-overexpressed cells (Figure 6a and b). Colony formation number and relative cell viabilities of HCT116 and SW480 cells in 3 days were overall lowered with miR-197-3p transfection than control transfection (Figure 6c–e), whereas these inhibitions were significantly diminished with co-transfection of the DKC1 vector (Figure 6c–e). Overexpressing miR-197-3p via mimic could arrest cell cycle progression and induce apoptosis in HCT116 and SW480 cells, as described by the promotion of G0/G1 cells and apoptosis rate, as well as the depression of S cells (Figure 6f–h). Nevertheless, restoring DKC1 via vector attenuated the effects of miR-197-3p overexpression on cell cycle distribution and apoptosis (Figure 6f–h). Transwell-migrated cells and invaded cells were reduced in miR-197-3p-expressed HCT116 and SW480 cells and were then recovered in cells co-expressing miR-197-3p and DKC1 (Figure 6i–k). In addition, miR-197-3p transfection aggravated L-OHP-evoked cell viability inhibition in HCT116 and SW480 cells, which was mitigated in the co-presence of the DKC1 vector (Figure 6l and m). These results demonstrated that reinforcing miR-197-3p suppressed malignant behaviors of CC cells and L-OHP resistance by inhibiting DKC1.

### 3.7 Depleting circ-FLI1 blocked tumorigenicity of CC cells by regulating miR-197-3p and DKC1

**Figure 7:**
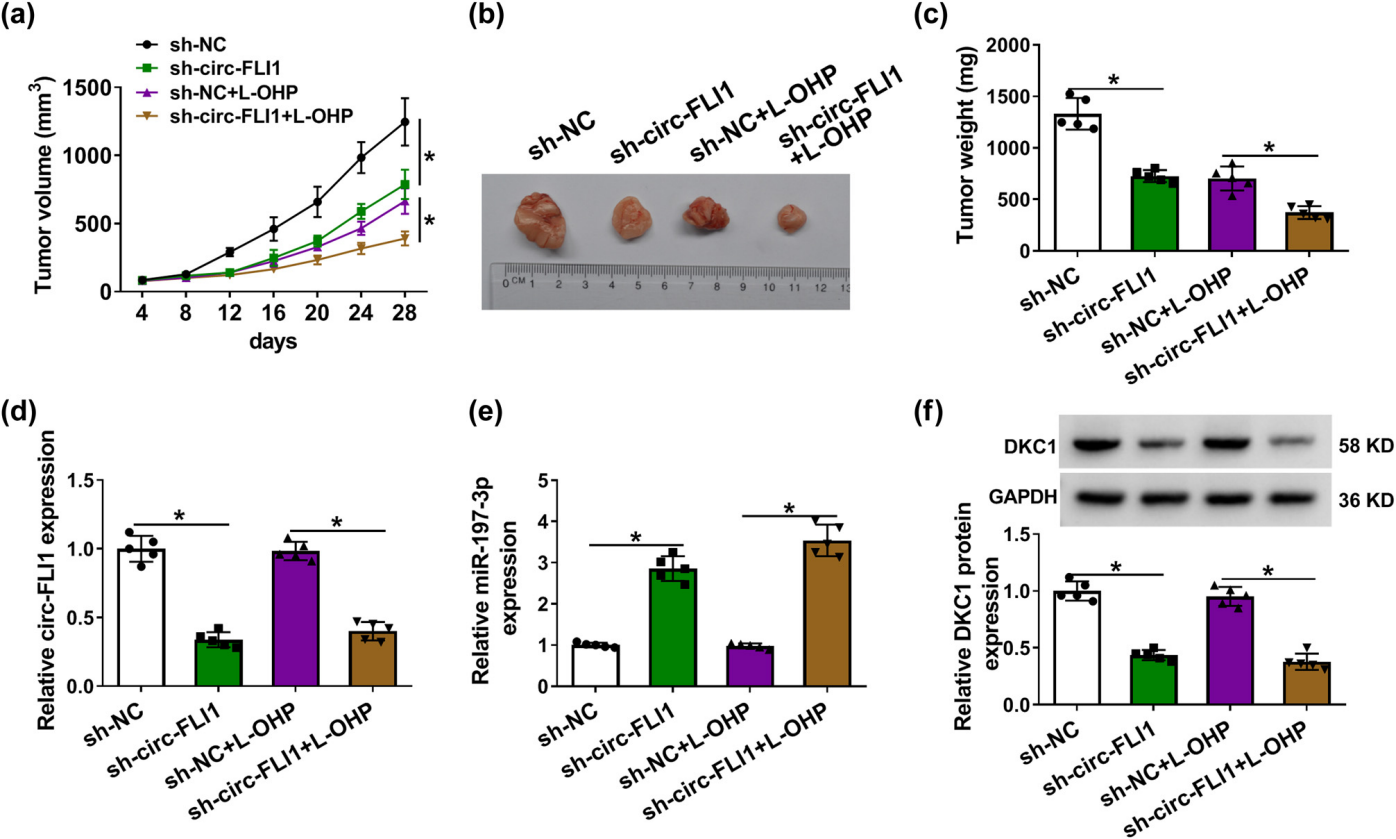
Depleting circ-FLI1 blocked tumorigenicity of CC cells. (a–f) Nude mice injected with sh-NC or sh-circ-FLI1-transfected HCT116 cells were divided into L-OHP and mock groups (*n* = 6/group). (a) Tumor volume was monitored every 4 days after cell transplantation. (b and c) Tumors were photographed and weighted after 28 days. (d and e) RT-qPCR detected relative circ-FLI1 and miR-197-3p expression and (f) western blotting detected relative DKC1 protein expression in xenograft tumor tissues. *P < 0.05.

The tumorigenicity experiment was carried out in nude mice, and sh-circ-FLI1-expressing HCT116 cells resulted in a delayed xenograft tumor growth regardless of L-OHP treatment, as evidenced by the decline of tumor volume and weight (Figure 7a–c). Moreover, the expression of circ-FLI1 was downregulated in xenograft tumor tissues and allied with higher miR-197-3p and lower DKC1 (Figure 7d–f). These *in vivo* data displayed that interfering circ-FLI1 caused tumor growth inhibition of CC by regulating miR-197-3p and DKC1.

## 4 Discussion

Recently, circRNA circ_0032833 was declared to regulate drug resistance, apoptosis, colony formation, and metastasis in 5-FU, leucovorin, and L-OHP (FOLFOX)-resistant CC cells via sponging miR-125-5p [24]. FLI1 was hypermethylated in colorectal adenomas and carcinomas [25], and plasma DNA methylation of FLI1 showed great clinical relevance in differing CC subtypes and tumor stages [26]. CircRNAs from exons of FLI1 could function as novel oncogenic drivers in lung and breast cancers [27,28]. Here, we demonstrated the role and molecular mechanism of circ-FLI1 in the malignant development of CC cells and L-OHP resistance.

Our data showed that circ-FLI1, as one exonic circRNA from FLI1, was abnormally upregulated in CC patients’ tumors and cells in a manner of RNase R resistance, and its expression was discovered in the cytosol. This dysregulation and subcellular localization of circ-FLI1 implied that it possessed the potential of being a regulator and miRNA sponge. Similarly, Ye et al. [8] detected an upregulation of circ-FLI1 in plasma samples of CC patients, and plasma circ-FLI1 had diagnostic value in CC. Here, tissue circ-FLI1 was associated with tumor size, TNM stage, distant metastasis, and unfavorable overall survival. Bioinformatics analyses and function annotation predicted that circ-FLI1 was abundant in miRNA-binding sites, and its function was mainly cancer-related [8]. Therein, we found that interfering with circ-FLI1 could inhibit colony formation, cell proliferation, and migration/invasion of CC cells and induce cell cycle arrest and apoptosis and L-OHP toxicity. Moreover, circ-FLI1 silencing could restrain xenograft tumor growth of CC cells in nude mice in both the L-OHP treatment group and the vehicle group. Previously, Zhang et al. [29] indicated circHIPK3 as a chemoresistant gene in CC by modulating L-OHP-induced cell viability inhibition, apoptosis, and autophagy according to gain-of-function assays in CC cells (HCT116 and HT29) and loss-of-function assays in the corresponding L-OHP-resistant cells. Furthermore, research investigations had demonstrated that certain ncRNAs, including circRNA and miRNA, were implicated in promoting and supporting chemoresistance of CC via different mechanisms [30]. In this pilot study, we noticed the association between circ-FLI1 and L-OHP toxicity in CC cells and xenograft tumors. However, the chemoresistant effects of circ-FLI1 in SW480, HCT116, SW480/L-OHP, and HCT116/L-OHP cells were not further determined.

Molecularly, we predicted and validated the target binding between miR-197-3p and either circ-FLI1 or functional gene DKC1. Besides, circ-FLI1 could modulate the expression of miR-197-3p-targeted DKC1, suggesting a competing endogenous RNA (ceRNA) regulatory model underlying circ-FLI1. Accidently, the above-mentioned expression model, cellular functions, and ceRNA mechanism of circ-FLI1 had overall been reported in acute myeloid leukemia [31]. However, this present study seemed to be a pilot study for the involvement of circ-FLI1 in chemoresistance.

According to our study, miR-197-3p was downregulated in CC tumors and cells, and its expression in CC patients was inversely and linearly correlated with circ-FLI1 predicting poor prognosis in the clinic. This finding was consistent with the previous study [32]. Moreover, Xu et al. [33] considered a 7-miRNA expression signature including miR-197-3p as an independent prognostic factor in colon adenocarcinoma patients. Furthermore, miR-197-3p expression was further downregulated in CC cells after 5-FU or L-OHP treatment [34]. Functionally, miR-197-3p might antagonize L-OHP resistance and malignant proliferation and migration/invasion of CC cells. In addition, its expression was decreased in 5-FU-resistant CC cells [35], and re-expressing miR-197-3p sensitized 5-FU resistance-acquired CC cells to 5-FU by suppressing cell proliferation and colony formation, enhancing apoptosis [35]. However, one evidence claimed that miR-197-3p could suppress primary 5-FU resistance in CC cells but did not influence CC cell proliferation and cell cycle [15]. All in all, these outcomes indicated a close association between miR-197-3p and chemoresistance in FOLFOX therapy in CC.

DKC1 is highly conserved and located at Xq28 [36]. Dyskerin is a key constituent of telomerase, and DKC1 maintains telomerase activity [37]. In this study, we observed an upregulation of DKC1 in CC patients’ tumors and cells, and its downregulation was contributing to the malignant behaviors of CC cells and L-OHP resistance. Notably, DKC1 upregulation had already been well reported in CC tissues [10,36,38], and DKC1 was considered as one diagnostic and prognostic factor for patients with CC [10]. Moreover, promoting the role of DKC1 had also been functionally validated in CC cell migration/invasion [10]. Thus, the functional role of DKC1 in CC cells was advanced in the prospect of L-OHP resistance herein.

In conclusion, the circ-FLI1/miR-197-3p/DKC1 ceRNA axis was underlying the tumorigenesis and development of CC cells and L-OHP resistance. Inhibiting circ-FLI1 might be a therapeutic approach for the treatment of CC.

## Appendix

Target screening results. All the computational targets of circ-FLI1 and miR-197-3p are according to different miRNA prediction databases.

**Table A1:** Correlation between circ-FLI1 and clinicopathological characteristics of patients with CC

Parameters	Case	circ-FLI1 expression		^a^ *P* value
		High (*n* = 28)	Low (*n* = 28)	
Age				
<60	31	17	14	0.420
≥60	25	11	14	
Gender				
Female	26	15	11	0.284
Male	30	13	17	
Tumor size (cm)				
<5	37	15	22	0.048*
≥5	19	13	6	
TNM stage				
I–II	38	15	23	0.022*
III	18	13	5	
Lymph node metastasis				
N0	32	18	14	0.280
N1–2	24	10	14	
Distant metastasis				
Absent	42	17	25	0.014*
Present	14	11	3	
Histological grade				
Well/moderately	31	13	18	0.179
Poorly	25	15	10	

CC patients were divided into high and low groups according to the median of circ-FLI1 expression in 56 CC tumors. **P* < 0.05, ^a^Chi-square test.

**Table A2:** The RT-qPCR primers

	Primers
circ-FLI1	Forward: CCACCCTCTACAACACGGAA
	Reverse: CCGACAGAGCCTCCTTCTTT
FLI1	Forward: TTAAGGAGGCTCTGTCGGTG
	Reverse: GAGGGGGTTGATCTTGTGGG
miR-197-3p	Forward: TTCACCACCTTCTCCAC
	Reverse: GAACATGTCTGCGTATCTC
DKC1	Forward: TCGGAAGTGGGGTTTAGGTC
	Reverse: GGCAGACTCACTGTAGTCAACAT
GAPDH	Forward: GACAGTCAGCCGCATCTTCT
	Reverse: GCGCCCAATACGACCAAATC
U6	Forward: GCTTCGGCAGCACATATACTAAAAT
	Reverse: CGCTTCACGAATTTGCGTGTCAT
